# Metabolic syndrome in antiphospholipid syndrome versus rheumatoid arthritis and diabetes mellitus: Association with arterial thrombosis, cardiovascular risk biomarkers, physical activity, and coronary atherosclerotic plaques

**DOI:** 10.3389/fimmu.2022.1077166

**Published:** 2023-01-09

**Authors:** Eleana Bolla, Nikolaos Tentolouris, Petros P. Sfikakis, Maria G. Tektonidou

**Affiliations:** ^1^ Rheumatology Unit, First Department of Propaedeutic Internal Medicine, Joint Academic Rheumatology Program, School of Medicine, National and Kapodistrian University of Athens, Laiko General Hospital, Athens, Greece; ^2^ Diabetes Center, First Department of Propaedeutic Internal Medicine, School of Medicine, National and Kapodistrian University of Athens, Laiko General Hospital, Athens, Greece

**Keywords:** antiphospholipid syndrome, metabolic syndrome, cardiovascular disease, atherosclerosis, thrombo-inflammation, cardiovascular risk factors, rheumatoid arthritis, diabetes mellitus

## Abstract

**Background:**

Cardiovascular disease (CVD) is the foremost cause of morbidity and deaths in antiphospholipid syndrome (APS), driven by thrombo-inflammation and atherothrombosis mechanisms. Metabolic syndrome (MetS) is a proinflammatory and prothrombotic state characterized by increased CVD risk. We aimed to evaluate the prevalence of MetS in APS patients compared to rheumatoid arthritis (RA) and diabetes mellitus (DM) and its associations with clinical and laboratory patient characteristics and vascular ultrasound (US) markers of subclinical atherosclerosis.

**Methods:**

We included 414 patients in our study: 138 patients with APS (median age: 44.9 years, females 70%) and matched 1:1 for age and sex RA and DM subjects. Three sets of criteria were used for MetS diagnosis: Joint Interim Statement (JIS), International Diabetes Federation (IDF) and modified National Cholesterol Education Program Adult Treatment Panel III (NCEP-ATPIII). The demographic, clinical and laboratory characteristics of all participants were recorded and carotid and femoral US was performed in patients with APS. Multivariate regression models were applied.

**Results:**

Prevalence of MetS was 23.9%, 23.2%, 20.3% (based on JIS, IDF, modified NCEP-ATPIII criteria, respectively) in APS versus 17.4%, 17.4%, 13% in RA (p=0.181, p=0.231, p=0.106, respectively), and 44.2%, 44.2%, 40.6% in DM patients. In multivariate analysis, patients with systemic lupus erythematosus- related APS had an approximately 2.5-fold higher risk of MetS versus RA patients. MetS in APS was independently associated with arterial thrombosis (Odds ratio 3.5, p=0.030). Odds ratio for MetS was 1.16 for each one unit increase in C-reactive protein levels according to JIS and IDF criteria, and 1.49 and 1.47 for each one unit increase in uric acid levels using the IDF and modified NCEP-ATPIII models, respectively. APS patients with atherosclerotic carotid plaques had 4 to 6.5-fold increased risk of MetS. Odds for MetS were decreased by 26% with an increase in physical activity by one hour per week.

**Conclusions:**

MetS is present in approximately one-fourth of APS patients at a comparable prevalence to that observed in patients with RA. MetS in APS is associated with arterial thrombosis, cardiovascular risk biomarkers, physical activity, and subclinical atherosclerosis, supporting its role in cardiovascular risk stratification and management in APS.

## 1 Introduction

Antiphospholipid syndrome (APS) is a systemic autoimmune disease, affecting mostly young adults, which is characterized by a wide spectrum of vascular and obstetric manifestations and the constant presence of antiphospholipid antibodies (aPL) (1). Cardiovascular disease (CVD), mainly in the form of stroke and myocardial infarction, is a leading cause of morbidity and mortality in APS (2). Innate and adaptive immune response dysregulation and an aPL- and traditional risk factors-mediated endothelial inflammation and damage play a major role in CVD pathogenesis in APS (3–8).

Metabolic syndrome (MetS) represents a constellation of interconnected cardiovascular risk factors (CVRFs), namely hypertension, abdominal obesity, insulin resistance and hyperglycemia, as well as elevated triglycerides (TGs) and low levels of high-density lipoprotein (HDL) cholesterol (9). It is currently recognized as an independent CVRF and its presence has been associated with an approximately two-fold increase in cardiovascular events in the general population (10). MetS shares common pathophysiologic pathways with APS involving a chronic low-grade systemic inflammation *via* pro-inflammatory cytokines production, macrophage recruitment, platelet activation, oxidative stress and endothelial dysfunction, insulin resistance, and free fatty acids production (11–14).

In the European League Against Rheumatism (EULAR) recommendations for the management of APS (15) and the EULAR recommendations for the management of cardiovascular risk in rheumatic and musculoskeletal diseases (RMDs) including Systemic Lupus Erythematosus (SLE) and APS (16), a thorough screening and control of traditional CVRFs was highlighted. Given that MetS is a cluster of modifiable CVRFs and shares endothelial damage pathways with APS, identification of its prevalence and any correlations with circulating cardiovascular biomarkers and clinical and subclinical CVD burden in APS, will help to improve CVD prevention measures in these patients.

Our goal was to evaluate the prevalence of MetS in APS using different sets of MetS diagnostic criteria and to examine its association with the clinical and laboratory features of the patients, as well as vascular ultrasound (US) markers of subclinical atherosclerosis. We also compared MetS prevalence in APS versus other rheumatic and non-rheumatic diseases of high CVD risk, such as rheumatoid arthritis (RA) and diabetes mellitus (DM).

## 2 Patients and methods

### 2.1 Study population

All eligible adult patients (≥18 years) who fulfilled the clinical and laboratory classification criteria for APS (1) and were followed at our Rheumatology Unit, were included in this cross-sectional study. Patients with APS were matched in a 1:1 ratio for age and sex with eligible patients with RA and DM followed in the Rheumatology and Diabetes Units of our Department, respectively. Exclusion criteria were prior atherosclerotic CVD events, concomitant DM (or RA, for patients with DM), acute illness (e.g., infectious disease), active malignancy, and pregnancy.

### 2.2 Recorded parameters

The following parameters were recorded at the time of the patients’ first visit at our department: age, sex, ethnicity, disease duration, APS type [primary APS (PAPS) or SLE-related APS (SLE-APS)], history of arterial and/or venous thrombosis, DM type for patients with DM, and traditional CVRFs, e.g. current smoking status and pack-years of smoking, blood pressure (BP) estimated as the average of three sequential readings taken 1 min apart after at least 10 min of rest (Microlife WatchBP Office, Microlife, Widnau, Switzerland), body mass index (BMI) (weight/height^2^), waist circumference (measured in cm), fasting total cholesterol (TC), low-density lipoprotein (LDL) and HDL cholesterol levels, fasting TGs levels, non-HDL levels (calculated by subtracting HDL from TC), physical activity level (measured in minutes of exercise per week), family history of coronary artery disease (CAD), and chronic kidney disease (CKD) (glomerular filtration rate <60 mL/min/1.73 m²). Additional laboratory tests included: C-reactive protein (CRP), glucose, uric acid (UA), haemoglobin A1c (HbA1c) levels, and aPL: anti-cardiolipin (aCL) antibodies (IgG or IgM isotype), anti-β2-glycoprotein I (anti-β2GPI) antibodies (IgG or IgM isotype) and lupus anticoagulant (LA). Positivity for aPL was defined based on the updated Sapporo criteria for APS (1). High titre aPL was defined as a titre greater than 4-fold of the upper normal limit in aCL or anti-β2GPI antibodies (IgG or IgM isotype). We also recorded disease-related medications including corticosteroids and cumulative prednisone dose, hydroxychloroquine and duration of its use, immunosuppressants and/or biologic agents, anticoagulants, antiplatelets, antihypertensives, lipid-lowering medications (statins, fibrates, ezetimibe, nicotinic acid, omega3 fatty acids supplements) and antidiabetic drugs.

Hypertension was defined as the use of antihypertensives or the average of three sequential office BP measurement >139/89 mmHg, and high-normal BP as the average of three sequential office systolic BP measurement of 130-139 mmHg and/or diastolic BP 85-89 mmHg in patients currently not on antihypertensives (17). Obesity was defined as BMI of at least 30 kg/m^2^, and abdominal obesity as a waist circumference of at least 80 cm in women and at least 94 cm in men (18). Dyslipidaemia in patients with APS and RA was defined as LDL ≥ 115 mg/dl and/or TGs ≥ 150 mg/dl and/or low HDL levels (<40 mg/dl in men and <50 mg/dl in women) and/or current use of lipid-lowering medication. Dyslipidemia was also assessed separately excluding the current use of lipid-lowering medications from the definition. The term atherogenic dyslipidemia was used to describe APS and RA patients with highly atherogenic lipid profile and it was defined as non-HDL ≥ 130 mg/dl and low HDL levels (for atherogenic dyslipidemia including non-HDL in the definition) and as TG ≥ 150mg/dl and low HDL levels (for atherogenic dyslipidemia including TGs in the definition) (19, 20).

For CVD risk classification in patients with APS and RA, we applied the Systemic Coronary Risk Evaluation (SCORE) (21) and its latest edition (SCORE2) to estimate 10-year risk of CVD. APS and RA patients were subsequently assigned to low, moderate and high CVD risk categories, based on the European Society of Cardiology (ESC) guidelines on CVD prevention of different years (17, 22, 23), according to the date of the patients’ US assessment. For CVD risk stratification in DM patients, we used the ESC guidelines for diabetes, prediabetes and cardiovascular diseases, developed in collaboration with the European Association for the Study of Diabetes (24), according to the time of their first visit. Based on the above, DM patients with at least one CVRF (hypertension, obesity, dyslipidaemia: fasting TC ≥200 mg/dL, LDL ≥130 mg/dL, HDL <40 mg/dL for men/<45 mg/dL for women or use of lipid-lowering medication (25), current smoking and CKD) and/or target organ damage are classified as very-high CVD risk, and all the other DM patients as high-risk.

### 2.3 Definition of MetS

The main components of MetS, as mentioned above, include abdominal obesity, hypertension, hyperglycemia and atherogenic dyslipidemia. Since the first description of MetS, several diagnostic criteria have been proposed for its definition, with differences concerning mainly the type and number of parameters required for the diagnosis, and the thresholds used for each parameter. In our study, we used three different sets of diagnostic criteria to define MetS: 1) the updated Joint Interim Statement (JIS) proposed by the International Diabetes Federation (IDF) Task Force on Epidemiology and Prevention; National Heart, Lung, and Blood Institute (NHLBI); American Heart Association (AHA); World Heart Federation; International Atherosclerosis Society; and International Association for the study of Obesity (26), 2) the IDF criteria (27) and 3) the National Cholesterol Education Program Adult Treatment Panel III (NCEP-ATPIII) criteria modified by AHA/NHLBI (25, 28). The above-mentioned criteria and the respective thresholds for each parameter are summarized in **Supplementary Table 1**.

### 2.4 Vascular US and outcome measures

Vascular US was performed in all APS patients by a single experienced operator. The near and far walls of the carotid bulbs, internal carotid arteries, common carotid arteries and common femoral arteries, bilaterally, were examined for the presence of atherosclerotic plaques using a 14-MHz multi-frequency linear transducer attached to a high-resolution B-mode US device (Vivid 7 Pro, GE Healthcare^®^). Plaques were defined as intima-media thickness (IMT) ≥1.5 mm or a focal thickening that encroaches ≥0.5 mm or 50% of the surrounding IMT into the arterial lumen.

### 2.5 Statistical analysis

Data are reported as median and interquartile range (IQR) (not normally distributed data), or when appropriate, as absolute number and relative frequency (percentage). To assess differences in patient characteristics, we applied Mann-Whitney U test (deviation from normality) for quantitative variables and Pearson’s χ^2^ or Fisher’s exact tests for qualitative variables. Pearson’s χ^2^ test was used to compare MetS prevalence between participant groups.

We applied multiple logistic regression models using the presence of MetS in APS patients as the outcome variable. All tested variables with a p-value<0.2 from the univariable logistic regression analysis were included in the initial multivariate logistic regression model. The backward elimination algorithm, based on which the variable with the highest p-value is removed in each step, along with clinical considerations, were used to derive the final multiple regression model (Supplementary ‘Backward elimination algorithm results’) resulting in three multivariate regression models, one for each definition of MetS used in the study, as the outcome variable. The final models included age, sex, arterial thrombosis, CRP and UA levels, high titre of anti-β2GPI antibodies of IgM isotype, presence of carotid atherosclerotic plaques, physical activity and current use of corticosteroids as independent variables. To further investigate the association of MetS (diagnosed based on the above three definitions) with different patient groups, we applied multiple regression models including an indicator variable with four levels denoting the participant group (1: RA, 2: PAPS, 3: SLE-APS, 4: DM). The outcome variable was the presence of MetS and the other independent variables in these models included age, sex, disease duration, pack-years of smoking, physical activity and LDL levels. A p-value <0.050 was considered statistically significant. STATA software (V.13.0, College Station, Texas, USA) was used for all statistical analyses.

## 3 Results

### 3.1 Baseline characteristics

A total of 138 patients with APS [85 with PAPS, 53 with SLE-APS, female 70.29%, median age 44.9 years (IQR: 36-53 years), all white Europeans] were included in the study, matched 1:1 for age and sex with RA and DM patients. Sixty three percent of DM patients had type I DM and 37% had type II DM [median HbA1c: 7.2% (IQR 6.7-8%)]. Basic characteristics of the three groups are shown in **Table 1**. The aPL profile and vascular US characteristics of APS patients are shown in **Table 2**. Atherosclerotic plaques at any site were present in 34.65% of APS patients, while 24.41% of patients had carotid plaques, and 21.26% had femoral artery plaques (**Table 2**).

**Table 1:** Patient characteristics in the APS, RA and DM patient groups.

Parameters	APS patients (excluding APS patients with known DM) (n=138)	Matched DM patients (n=138)	Matched RA patients (n=138)	p-value*
Age [median (IQR)]	44.9 (36–53)	44 (36-55)	45.5 (37-53)	0.902
Female sex [N, (%)]	97/138 (70.29)	97/138 (70.29)	97/138 (70.29)	1.000
Primary APS [N, (%)]	85/138 (61.59)	–	–	–
Disease duration (years) [median (IQR)]	6 (1.6-14)	12.5 (5-23)	7 (2-16)	0.000
Family history of CAD [N, (%)]	17/138 (12.32)	15/138 (10.87)	16/138 (11.59)	0.932
CKD [N, (%)]	9/138 (6.52)	12/138 (8.70)	2/138 (1.45)	0.018
Smoking current [N, (%)]	43/138 (31.16)	41/138 (29.71)	31/138 (22.46)	0.225
Smoking (pack-years) [median, (IQR)]	2.5 (0-20)	0.1 (0-20)	0 (0-15)	0.311
Exercise (min/week) [median, (IQR)]	0 (0-180)	120 (0-240)	0 (0-120)	0.001
BMI (kg/m^2^) [median, (IQR)]	27.1 (23.7-31.4)	28 (23.5-32.5)	26.3 (23.5-28.6)	0.031
Waist circumference (cm) [median, (IQR)]	90 (80-100)	93 (79-105)	90 (77-98)	0.127
Obesity [N, (%)]	45/138 (32.61)	49/138 (35.51)	27/138 (19.57)	0.008
Abdominal obesity [N, (%)]	92/138 (66.67)	87/138 (63.04)	89/138 (64.49)	0.818
SBP (mmHg) [median, (IQR)]	123 (115-132)	122 (113-138)	126.5 (116-137)	0.297
DBP (mmHg) [median, (IQR)]	74.5 (69-80)	74 (68-79)	79 (73-85)	0.000
High-normal BP [N, (%)]	21/96 (21.88)	13/100 (13.00)	30/108 (27.78)	0.032
Hypertension [N, (%)]	54/138 (39.13)	49/138 (35.51)	48/138 (34.78)	0.724
Antihypertensives, current use [N, (%)]	42/138 (30.43)	38/138 (27.54)	30/138 (21.74)	0.250
Cholesterol (mg/dl) [median, (IQR)]	179 (156-206)	183 (163-210)	203 (166-236)	0.002
LDL (mg/dl) [median, (IQR)]	100.5 (82-128)	105 (92-126)	119 (86-141)	0.027
HDL (mg/dl) [median, (IQR)]	52 (43-65)	56 (43-65)	62 (51-76)	0.000
Triglycerides (mg/dl) [median, (IQR)]	95 (71-135)	72.5 (54-118)	77 (65-115)	0.006
non-HDL (mg/dl) [median, (IQR)]	122 (105-152)	127.5 (108-149)	136 (105-162)	0.098
Lipid-lowering medication, current use [N, (%)] **	25/138 (18.12)	37/138 (26.81)	12/138 (8.70)	0.000
• Statins, current use [N, (%)] • Fibrates, current use [N, (%)] • Ezetimibe, current use [N, (%)] • Omega-3 supplements, current use [N, (%)]	24/138 (17.39) 1/138 (0.72) 1/138 (0.72) 0 (0)	37/138 (26.81) 4/138 (2.90) 2/138 (1.45) 4/138 (2.90)	10/138 (7.25) 0 (0) 1/138 (0.72) 1/138 (0.72)	0.000 0.133 1.000 0.133
Dyslipidaemia including the use of lipid-lowering medication in the definition [N, (%)] ***	89/138 (64.49)	82/138 (59.42)	93/138 (67.39)	0.378
Dyslipidaemia excluding the use of lipid-lowering medication from the definition [N, (%)] ***	82/138 (59.42)	69/136 (50.74)	86/138 (62.32)	0.131
Atherogenic dyslipidemia (including Triglycerides in the definition) [N, (%)]	11/138 (7.97)	–	4/138 (2.90)	0.108
Atherogenic dyslipidemia (including non-HDL in the definition) [N, (%)]	22/138 (15.94)	–	8/138 (5.80)	0.007
SCORE class [N, (%)] • Low/moderate risk • High risk • Very high risk	127/138 (92.03) 8/138 (5.80) 3/138 (2.17)	0 (0) 28/138 (20.29) 110/138 (79.71)	125/138 (90.58) 9/138 (6.52) 4/138 (2.90)	0.000
Glucose (mg/dl) [median, (IQR)]	89 (82-96)	128.5 (92-169.5)	90 (82-99)	0.000
CRP (mg/l) [median, (IQR)]	2.35 (0.60-5.06)	–	–	–
UA (mg/dl) [median, (IQR)]	5 (4.0 - 6.1)	–	–	–
Arterial thrombosis [N, (%)]	70/138 (50.72)	–	–	–
Venous thrombosis [N, (%)]	80/138 (57.97)	–	–	–
Hydroxychloroquine, current use [N, (%)]	56/138 (40.58)	–	10/138 (7.25)	0.000
Hydroxychloroquine use duration (months) [median, (IQR)]	1.5 (0-27)	–	–	–
Cortisone, current use [N, (%)]	41/138 (29.71)	–	83/138 (60.14)	0.000
Cumulative prednisone dose (mg) [median, (IQR)]	0 (0-6125)	–	–	–
Aspirin, current use [N, (%)]	57/138 (41.30)	12/138 (8.70)	1/138 (0.72)	0.000
Anticoagulants, current use [N, (%)]	102/138 (73.91)	5/138 (3.62)	4/138 (2.90)	0.000
Immunosuppressive drugs, current use [N, (%)]	23/138 (16.67)	–	89/138 (64.49)	0.000
Biologic factor treatment [N, (%)]	1/138 (0.72)	–	67/138 (48.55)	0.000

* p-values refer to the comparisons between the three patient groups in the table.

** Some patients using currently more than one type of lipid-lowering medication.

*** Dyslipidaemia definition according to the patient group.

APS, Antiphospholipid syndrome; RA, Rheumatoid arthritis; DM, Diabetes mellitus; CAD, Coronary artery disease; CKD, Chronic kidney disease; BMI, Body mass index; BP, Blood pressure; SBP, Systolic blood pressure; DBP, Diastolic blood pressure; LDL, Low-density lipoprotein; HDL, High-density lipoprotein; SCORE, Systemic Coronary Risk Evaluation; CRP, C-reactive protein; UA, Uric acid.

# There are missing data for some of the parameters in the table above; the denominator is the total number of available values for each parameter.

**Table 2:** Antiphospholipid antibody profile and vascular ultrasound characteristics of APS patients (excluding APS patients with known diabetes mellitus).

Antiphospholipid antibody profile	
Anti-cardiolipin IgG positivity [N, (%)]	92/138 (66.67)
High-titre anti-cardiolipin IgG [N, (%)]	40/138 (28.99)
Anti-cardiolipin IgM positivity [N, (%)]	72/138 (52.17)
High-titre anti-cardiolipin IgM [N, (%)]	31/138 (22.46)
Anti-β2-glycoprotein I IgG positivity [N, (%)]	65/138 (47.10)
High-titre anti-β2-glycoprotein I IgG [N, (%)]	31/138 (22.46)
Anti-β2-glycoprotein I IgM positivity [N, (%)]	56/138 (40.58)
High-titre anti-β2-glycoprotein I IgM [N, (%)]	18/138 (13.04)
Lupus anticoagulant positivity [N, (%)]	97/138 (70.29)
High-titre antiphospholipid antibody [N, (%)]	66/138 (47.83)
Triple antiphospholipid antibody positivity [N, (%)]	61/138 (44.20)
Antiphospholipid antibody profile	
Atherosclerotic plaques at any site [N, (%)]	44/127 (34.65)
Atherosclerotic plaques at the carotid arteries [N, (%)]	31/127 (24.41)
Atherosclerotic plaques at the femoral arteries [N, (%)]	27/127 (21.26)
Total number of atherosclerotic plaques [median, (IQR)]	0 (0-1)

APS, Antiphospholipid syndrome.

# There are missing data for some of the parameters in the table above; the denominator is the total number of available values for each parameter.

Among the three patient groups, TG levels were higher and HDL levels were lower in APS patients than the other two groups (p=0.006 and p<0.001, respectively), and obesity was more prevalent in APS and DM patients compared to RA patients (p=0.008). DM patients had longer disease duration than APS and RA patients (p<0.001) and higher exercise levels and current use of lipid-lowering medications (p=0.001 and p<0.001, respectively). There were no significant differences between the three groups in the current smoking status and pack-years of smoking, hypertension, abdominal obesity, and family history of CAD.

### 3.2 MetS prevalence and associations in three patient groups

**Figure 1:**
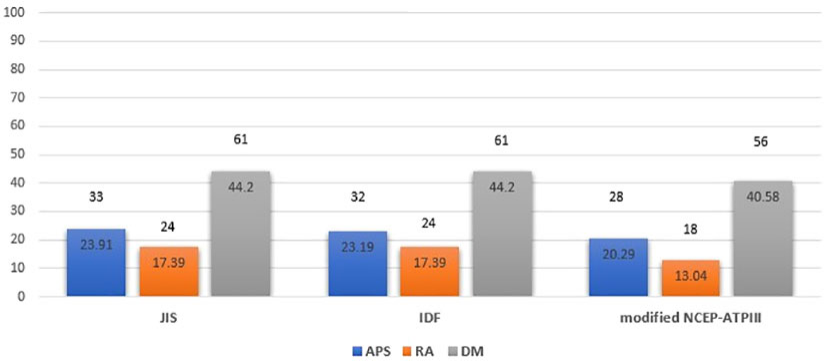
Metabolic syndrome prevalence in APS, RA and DM patient groups based on JIS, IDF and modified NCEP-ATPIII criteria (Percentages and absolute numbers are noted) # Comparison for APS versus RA patients: p=0.181, p=0.231, p=0.106 based on JIS, IDF and modified NCEP-ATPIII criteria respectively. Comparison for APS versus DM patients: p<0.001 for all criteria. Comparison for RA versus DM patients: p<0.001 for all criteria. Comparison for APS versus RA versus DM patients: p<0.001 for all criteria. APS, Antiphospholipid syndrome; RA, Rheumatoid arthritis; DM, Diabetes mellitus; JIS, Joint Interim Statement; IDF, International Diabetes Federation; NCEP-ATPIII, National Cholesterol Education Program Adult Treatment Panel III.

Based on JIS, IDF and modified NCEP-ATPIII criteria, a comparable prevalence of MetS was detected between patients with APS (23.91%, 23.19% and 20.29%, respectively) and patients with RA (17.39%, 17.39% and 13.04%, p=0.181, p=0.231, p=0.106, respectively), with the highest prevalence in the age/sex-matched DM group (44.2%, 44.2% and 40.58%, respectively). (**Figure 1**) Among APS patients, MetS was present in 23.53% versus 24.53%, 22.35% versus 24.53% and 20% versus 20.75%, in PAPS versus SLE-APS patients respectively, based on JIS, IDF and modified NCEP-ATPIII criteria, respectively.

In multiple regression analysis including the participant group as an independent variable, patients with SLE-APS had an approximately 2.5-fold higher risk of MetS versus RA patients. MetS presence using the JIS, IDF and modified NCEP-ATPIII models was also independently associated with age (p<0.001 in all models), disease duration (p=0.024, p=0.023, p=0.002, respectively), physical activity (p=0.014, p=0.017, p=0.021, respectively), pack-years of smoking (p=0.009, p=0.005, p=0.002, respectively) and LDL levels (p=0.015, p=0.011, p=0.043, respectively) (**Table 3**).

**Table 3:** Disease conferred risk for the presence of metabolic syndrome in patients with PAPS, SLE-APS and DM based on JIS, IDF and modified NCEP-ATPIII criteria (in multivariate regression models).

Parameters	JIS criteria	IDF criteria	Modified NCEP- ATPIII criteria
**Participant group** • **RA (reference category)** • **PAPS** • **SLE-APS** • **DM**	OR 1.803 (95% CI 0.823,3.916), p=0.137 OR 2.246 (95% CI 0.933,5.405), p=0.071 OR 9.041 (95% CI 4.365,18.729), p=0.000	OR 1.666 (95% CI 0.762,3.643), p=0.201 OR 2.211 (95% CI 0.919,5.322), p=0.077 OR 9.020 (95% CI 4.353,18.692), p=0.000	OR 2.183 (95% CI 0.925,5.152), p=0.075 OR 2.735 (95% CI 1.048,7.137), p=0.040 OR 13.212 (95% CI 5.862,29.782), p=0.000
**Age (years)**	OR 1.086 (95% CI 1.060,1.112), p=0.000	OR 1.084 (95% CI 1.058,1.111), p=0.000	OR 1.091 (95% CI 1.063,1.119), p=0.000
**Female sex**	OR 0.909 (95% CI 0.489,1.690), p=0.763	OR 0.978 (95% CI 0.524,1.827), p=0.944	OR 1.239 (95% CI 0.623,2.462), p=0.541
**Disease duration (years)**	OR 0.969 (95% CI 0.942,0.996), p=0.024	OR 0.968 (95% CI 0.941,0.995), p=0.023	OR 0.951 (95% CI 0.922,0.982), p=0.002
**Physical activity (min/week)**	OR 0.998 (95% CI 0.996,1.000), p=0.014	OR 0.999 (95% CI 0.996,1.000), p=0.017	OR 0.998 (95% CI 0.996,1.000), p=0.021
**Pack – years of smoking**	OR 1.021 (95% CI 1.005,1.037), p=0.009	OR 1.022 (95% CI 1.007,1.038), p=0.005	OR 1.027 (95% CI 1.010,1.043), p=0.002
**LDL levels (mg/dl)**	OR 1.010 (95% CI 1.002,1.017), p=0.015	OR 1.010 (95% CI 1.002,1.018), p=0.011	OR 1.008 (95% CI 1.000,1.017), p=0.043

RA, Rheumatoid arthritis; PAPS, Primary antiphospholipid syndrome; SLE-APS, Systemic lupus erythematosus- related antiphospholipid syndrome; DM, Diabetes mellitus; CI, Confidence intervals; JIS, Joint Interim Statement; IDF, International Diabetes Federation; NCEP-ATPIII, National Cholesterol Education Program Adult Treatment Panel III; LDL, Low-density lipoprotein.

### 3.3 MetS prevalence and associations in APS patients

Focusing only on patients with APS, we included in the analysis three additional APS patients with co-existent DM who were excluded from the analysis among the three patient groups (because DM was one of the comparison groups), resulting in a total of 141 patients in the APS group. In this case, MetS was present in 25.53%, 24.82% and 21.99% of APS patients, based on JIS, IDF and modified NCEP-ATPIII criteria, respectively.

**Figure 2:**
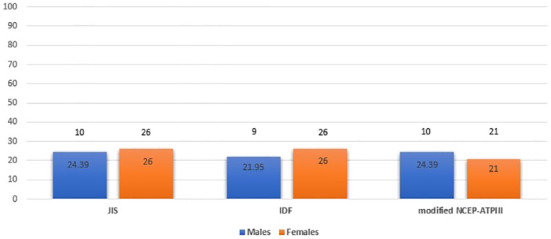
Metabolic syndrome prevalence in APS patients by sex (males and females) based on JIS, IDF and modified NCEP-ATPIII criteria (Percentages and absolute numbers are noted) APS, Antiphospholipid syndrome; JIS, Joint Interim Statement; IDF, International Diabetes Federation; NCEP-ATPIII, National Cholesterol Education Program Adult Treatment Panel III.

**Figure 3:**
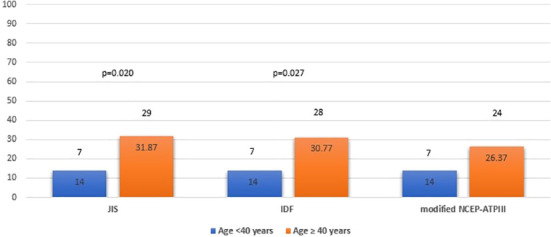
Metabolic syndrome prevalence in APS patients by age group (<40 years old and ≥ 40 years old) based on JIS, IDF and modified NCEP-ATPIII criteria (Percentages and absolute numbers are noted) APS, Antiphospholipid syndrome; JIS, Joint Interim Statement; IDF, International Diabetes Federation; NCEP-ATPIII, National Cholesterol Education Program Adult Treatment Panel III.

We also examined potential differences in the prevalence of MetS by age and sex. MetS prevalence was comparable between male and female patients with APS, using the JIS, IDF and modified NCEP-ATPIII definition (24.39%, 21.95% and 24.39% for males versus 26%, 26% and 21% for females, respectively) (**Figure 2**) but significantly higher among APS patients aged ≥ 40 years than those < 40 years (31.87%, 30.77% and 26.37% versus 14%, 14% and 14%; p=0.020, p=0.027 and p=0.090, respectively) (**Figure 3**).

The results of the univariable logistic regression analysis for all tested variables are presented in **Supplementary Table 2**. In multivariate logistic regression models, we investigated the associations of MetS with clinical and laboratory parameters of APS and vascular US markers of subclinical atherosclerosis. We found significant associations with arterial thrombosis, CRP and UA levels, physical activity and presence of carotid atherosclerotic plaques, after controlling for age, sex, current use of corticosteroids and high titre of anti-β2GPI antibodies of IgM isotype based on backward elimination algorithm (**Table 4**). Interestingly, APS patients with arterial thrombosis had a 3.5-fold increased risk of MetS using the IDF criteria (p=0.030) with a trend in the JIS model [Odds ratio (OR) =2.76, p=0.062). Physical activity and CRP levels were independently associated with MetS using the JIS and IDF definitions, and the UA levels using the IDF and modified NCEP-ATPIII models. In particular, the odds for MetS were decreased by approximately 26% with an increase in physical activity by one hour per week in the JIS and IDF models. The OR for MetS was 1.16 for each one unit increase in CRP levels according to both JIS and IDF criteria. In the IDF and modified NCEP-ATPIII models, the ORs for MetS were 1.49 and 1.47, respectively for each one unit increase in UA levels. APS patients with atherosclerotic carotid plaques had 4 to 6.5-fold increased risk of MetS (OR=6.37, p=0.007; OR=4.69, p=0.024; and OR=4.15, p=0.033, using the JIS, IDF and modified NCEP-ATPIII models, respectively).

**Table 4:** Multivariate determinants for the presence of metabolic syndrome in APS patients based on JIS, IDF and modified NCEP-ATPIII criteria.

Parameters	JIS criteria	IDF criteria	Modified NCEP- ATPIII criteria
**Age (years)**	OR 0.976 (95% CI 0.929,1.026), p=0.340	OR 0.972 (95% CI 0.924,1.022), p=0.266	OR 0.980 (95% CI 0.932,1.030), p=0.417
**Female sex**	OR 0.997 (95% CI 0.260,3.829), p=0.997	OR 1.465 (95% CI 0.366,5.869), p=0.590	OR 0.742 (95% CI 0.193,2.853), p=0.664
**Arterial thrombosis**	OR 2.761 (95% CI 0.952,8.013), p=0.062	OR 3.399 (95% CI 1.129,10.235), p=0.030	OR 1.902 (95% CI 0.660,5.479), p=0.234
**CRP levels (mg/L)**	OR 1.162 (95% CI 1.033,1.307), p=0.012	OR 1.159 (95% CI 1.032,1.302), p=0.012	OR 1.107 (95% CI 0.994,1.233), p=0.063
**UA levels (mg/dL)**	OR 1.396 (95% CI 0.964,2.021), p=0.077	OR 1.487 (95% CI 1.016,2.176), p=0.041	OR 1.465 (95% CI 1.015,2.113), p=0.041
**Physical activity (min/week)**	OR 0.995 (95% CI 0.990,0.999), p=0.022	OR 0.995 (95% CI 0.991,1.000), p=0.034	OR 0.996 (95% CI 0.992,1.000), p=0.081
**Presence of carotid atherosclerotic plaques**	OR 6.367 (95% CI 1.658,24.454), p=0.007	OR 4.685 (95% CI 1.226,17.897), p=0.024	OR 4.151 (95% CI 1.122,15.359), p=0.033
**Current use of corticosteroids**	OR 2.164 (95% CI 0.713,6.563), p=0.173	OR 2.262 (95% CI 0.745,6.866), p=0.150	OR 2.861 (95% CI 0.931,8.793), p=0.067
**High titre anti-β2-glycoprotein I IgM**	OR 3.705 (95% CI 0.873,15.729), p=0.076	OR 4.069 (95% CI 0.945,17.522), p=0.060	OR 1.695 (95% CI 0.385,7.645), p=0.485

CI, Confidence intervals; JIS, Joint Interim Statement; IDF, International Diabetes Federation; NCEP-ATPIII, National Cholesterol Education Program Adult Treatment Panel III; CRP, C-reactive protein; UA, uric acid.

Finally, we also examined whether meeting the diagnostic criteria of MetS is associated with atherosclerotic plaque presence, following the multivariate regression analysis methodology described in the statistical analysis section. In these models, the presence of MetS based on JIS, IDF and modified NCEP-ATPIII criteria was significantly associated with the presence of atherosclerotic plaques at any site (OR 3.44, p=0.022; OR 2.92, p=0.050; OR 3.53, p=0.029, respectively) after adjusting for age, sex, APS type (PAPS or SLE-APS), pack-years of smoking, current use of statins and history of arterial and venous thrombosis.

## 4 Discussion

Our study showed that MetS is present in about one-fourth of APS patients and it is associated with arterial thrombosis, inflammation and cardiovascular biomarkers, physical activity levels and subclinical atherosclerosis, supporting the need for its rigorous assessment and management in APS. To our knowledge, this is the first study that compares the prevalence of MetS among patients with APS and other rheumatic and non-rheumatic conditions of high CVD risk, and the first study that examines any potential associations with clinical, laboratory and vascular US characteristics in APS.

CVD, especially myocardial infarction and stroke, represents a leading cause of death in APS, referring to 18.9% and 13.2% of deaths, respectively, in a large European cohort study of 1000 APS patients over a 10-year follow-up period (2). MetS is increasingly recognized as an independent predictor of CVD morbidity and mortality that implies substantial additional CVD risk beyond the sum of individual CVRF components (29). MetS is characterized by systemic inflammation, prothrombotic changes, endothelial dysfunction and accelerated atherosclerosis, sharing common pathogenetic mechanisms with APS. A growing body of evidence suggests that RMDs, especially SLE (30) and inflammatory arthritides (31–33), are characterized by increased prevalence of MetS, compared to healthy individuals, while recent meta-analyses showed that patients with SLE (OR=1.88 and OR=2.5, respectively, in 2 recent meta-analyses (34, 35), and RA (OR=1.44) (36) had high risk of MetS comparing to controls. MetS has been reported in 17 - 38% of APS patients in different studies with the use of various sets of diagnostic criteria (8, 37–40). This finding could be attributed to chronic inflammation and innate immune cell activation in these disorders, and a higher prevalence of traditional CVRFs. Data from the Greek APS registry (41) and recent data from our (42) and other groups (43) showed a comparable or higher prevalence of traditional CVRFs to that observed in age and sex-matched patients with RA or DM, such as obesity, smoking, hypertension and dyslipidaemia. In addition, in a previous case-control study conducted by our group, patients with PAPS accumulated more traditional CVRFs than age and sex-matched patients with DM (44).

We also found that MetS prevalence was significantly higher in patients aged ≥40 years than in younger patients. This finding is consistent with previous relevant studies in the general population worldwide (45–48) and in patients with SLE and RA (49, 50) indicating that MetS is more prevalent with advancing age. No significant difference was found in MetS prevalence between male and female patients with APS regardless of the diagnostic criteria used. Data on sex-related differences concerning MetS prevalence in the general population is relatively scarce and conflicting, although it is inferred that perimenopausal hormonal alterations and associated changes in body fat distribution, insulin sensitivity and lipid levels might determine an age-associated increased prevalence of MetS among women (51, 52).

Interestingly, we found a comparable frequency of MetS in APS with that observed in patients with RA, a disorder characterized by high systemic inflammation, a high prevalence of CVRFs and a substantial CVD burden. Importantly, MetS risk was higher in the SLE-APS subgroup versus RA patients. To date, there are only isolated studies comparing patients with SLE and RA with respect to MetS (53, 54) and no studies comparing APS and RA. In one of these studies, MetS prevalence was similar between 85 SLE and 107 RA patients included in the study (53). Santos et al. (54) reported that hypertension, hyperuricemia and low HDL levels were more prevalent in 100 SLE women than 98 RA women with similar mean age. A significantly higher prevalence of MetS was found in patients with DM vs both APS and RA patients in our study. This finding was expected based on the high prevalence of traditional CVRFs in these patients and the mere definition of MetS, which includes hyperglycemia and previously diagnosed DM as a diagnostic component.

Examining associations between MetS and laboratory and clinical CVD markers, we found that MetS was independently associated with CRP and UA as well as with arterial thrombosis, physical activity and atherosclerotic plaques, respectively. Various inflammation biomarkers have been measured in patients with MetS, of which CRP is the most well-characterized (55, 56). Evidence has shown that CRP levels are elevated in patients with MetS in both the general population (57, 58) and patients with RMDs, such as SLE (30, 59, 60) and RA (61, 62). Accordingly, our results in APS showed that an increase in CRP levels is independently associated with MetS presence, using JIS and IDF diagnostic criteria. This finding is of high importance, taking into consideration the well-established role of CRP in CVD risk assessment and its recognition as an independent predictor of cardiovascular events in MetS (55). Hyperuricemia was also found to be significantly associated with an increased risk for MetS in our study. These results are consistent with a previous case-control study by Rodrigues et al. (37) reporting significantly higher UA levels in APS patients with MetS than those without. Hyperuricemia, another increasingly recognized cardiovascular risk biomarker has been linked to MetS and its components, including insulin resistance and type II DM, hypertension, abdominal obesity and dyslipidemia, in various studies in the general population (63–67). The relationship between serum UA and markers of inflammation, endothelial dysfunction and subclinical atherosclerosis in the general population has also been extensively studied (68–71).

Concerning associations with clinical and subclinical CVD parameters, we found that APS patients with arterial thrombosis (mainly stroke and CAD) have a 3.5-fold higher risk for MetS, based on the IDF diagnostic criteria, and this finding was confirmed with a trend using the JIS definition. In a previous case-control study, researchers demonstrated that PAPS patients with MetS had a higher frequency of arterial events, strengthening the concept of synergistic effect of APS and MetS on endothelial dysfunction and atherothrombosis (37). Subclinical atherosclerosis is recognized as an early indicator of CVD burden in the general population. In a case-control study conducted by our group, we found that both patients with PAPS and SLE-APS had a nearly 2.5-fold risk of atherosclerotic plaques in carotid and/or femoral arteries compared to controls, and similar to DM patients, after adjusting for traditional CVRFs (44), and a comparable relative risk for plaque progression between patients with PAPS, SLE-APS and DM in a 3-year follow-up study (72). The present study examined for the first time the association between atherosclerotic plaque burden and the presence of MetS in APS patients. We showed that APS patients with carotid plaques have a 4 to 6.5-fold increased risk of MetS, confirmed by the three different diagnostic criteria used. Our results are in accordance with observational studies (73–78) and recent meta-analyses (79, 80) of population-based studies which showed that MetS is a risk factor for early carotid atherosclerosis in the general population. Consequently, these data support the role of MetS and vascular US examination in cardiovascular risk stratification and CVD prevention decision-making strategies in APS.

In the same line, we found that physical activity is associated with lower risk for MetS in all three disease groups including APS. This finding is expected, as it is widely known that regular exercise has a favorable effect on body weight, BP, lipid levels and insulin resistance, which are all components of MetS (17, 22, 23). It is also postulated that physical activity has anti-inflammatory effects as it has been negatively associated with high-sensitive CRP, interleukin (IL)-6, IL-18, and positively associated with the anti-inflammatory cytokine IL-10 (81).

The strengths of our study include the assessment for the first time of the following: 1) prevalence of MetS using three different sets of MetS diagnostic criteria in a large cohort of APS patients considering the rarity of the syndrome (representing the largest so far study examining MetS in APS); 2) associations between MetS and multiple clinical, laboratory and subclinical atherosclerosis parameters assessed simultaneously; 3) differences in MetS prevalence comparing patients with APS and age and sex-matched patients with rheumatic and non-rheumatic disorders of high CVD risk, such as RA and DM. However, prospective, multicentre studies are required to confirm our results. The study has some limitations. The group of DM controls is heterogeneous, since 63% of them had type I DM and 37% had type II DM, which may also account for some of the results e.g. the difference in disease duration among the groups.

In conclusion, our study showed that MetS is present in one-fourth of APS patients, a comparable prevalence to that observed in RA. MetS in APS is associated with arterial thrombosis, cardiovascular biomarkers and markers of inflammation, as well as subclinical atherosclerosis. Awareness of MetS among clinicians and patients with APS, as well as thorough screening and control of traditional CVRFs following similar CVD prevention measures to those implemented in other diseases of high CVD risk, could help to improve cardiovascular health in APS.

## Data availability statement

The raw data supporting the conclusions of this article will be made available by the authors, without undue reservation.

## Ethics statement

The study was approved by the hospital’s Institutional Review Board of Laiko General Hospital (Laiko Hospital Scientific Council; IRB number 7748). The patients/participants provided their written informed consent to participate in this study.

## Author contributions

EB: acquisition of data, analysis of data, drafting and critical revision of the manuscript. NT: acquisition of data, critical revision of manuscript. PS: interpretation of data, critical revision of manuscript. MT: conception and design of the study, acquisition of data, analysis and interpretation of data, drafting and critical revision of the manuscript, guarantor. All authors contributed to the article and approved the submitted version.

## Supplementary material

Supplementary Table 1 Criteria for the clinical diagnosis of metabolic syndrome based on different sets of diagnostic criteria

Supplementary Table 2 Univariate determinants for the presence of metabolic syndrome in APS patients based on JIS, IDF and modified NCEP-ATPIII criteria
