# Supplementary material for: Metabolic syndrome in antiphospholipid syndrome versus rheumatoid arthritis and diabetes mellitus: Association with arterial thrombosis, cardiovascular risk biomarkers, physical activity, and coronary atherosclerotic plaques
Source: Front Immunol. 2023 Jan 9;13:1077166. doi: 10.3389/fimmu.2022.1077166 (PMC9868803; doi:10.3389/fimmu.2022.1077166)
Supplement: Supplementary Table 1 — Criteria for the clinical diagnosis of metabolic syndrome based on different sets of diagnostic criteria [file DataSheet_1.docx]

**Supplementary material**

**Results**

**Backward elimination algorithm results:**

We applied multiple logistic regression models using the presence of metabolic syndrome (MetS) in antiphospholipid syndrome (APS) patients as the outcome variable, after performing univariable logistic regression analysis for each parameter. The results of the univariable regression models are presented in Supplementary Table 2. All tested variables with a p-value<0.2 according to the univariable logistic regression analysis were included in the initial multivariate logistic regression model. The backward elimination algorithm, based on which the variable with the highest p-value is removed in each step, along with clinical considerations, were used to derive the final multivariate regression model. Sex and age were considered clinically relevant and were therefore included in all models regardless of the p-value. Based on the above, the initial models included the following parameters: age, sex, pack-years of smoking, exercise, total cholesterol (TC) and low-density lipoprotein (LDL) cholesterol levels, current use of statins, aspirin, anticoagulants, hydroxychloroquine and glucocorticoids, Systemic Coronary Risk Evaluation (SCORE) classification, C-reactive protein and uric acid levels, presence of chronic kidney disease (CKD), history of arterial thrombosis, high titre of anti-β2- glycoprotein I IgG antibodies, anti-β2-glycoprotein I IgM antibody positivity and high titre of anti-β2- glycoprotein I IgM antibodies, atherosclerotic plaques at any site and at carotid and femoral arteries, and the total number of plaques. The following parameters, which were statistically significant in the univariable analysis, were eliminated from the final multiple regression models, due to higher p-values than other variables and statistical insignificance in the next steps of the backward elimination process: pack-years of smoking, TC and LDL levels, current use of statins and hydroxychloroquine, SCORE classification, atherosclerotic plaques at any site and total number of plaques. In addition, the following parameters with a p-value<0.2 in the univariable analysis, were further eliminated from the final multivariate models due to higher p-values than other variables and statistical insignificance, based on the backward elimination process: current use of aspirin and anticoagulants, presence of CKD, high titre of anti-β2-glycoprotein I IgG antibodies, anti-β2-glycoprotein I IgM antibody positivity and atherosclerotic plaques in the femoral arteries.

**Supplementary Table 1. Criteria for the clinical diagnosis of metabolic syndrome based on different sets of diagnostic criteria**

|  | **JIS criteria** | **IDF criteria** | **Modified NCEP-ATPIII criteria** |
| --- | --- | --- | --- |
| **Criteria required for the diagnosis** | Presence of any 3 out of 5 following criteria | Central obesity (waist circumference ≥80cm for Europid women and ≥94cm for Europid men) plus any two of the following criteria | Presence of any 3 out of 5 following criteria |
| **Waist circumference** | ≥80cm for women and ≥94cm for men (Mediterranean population) | - | ≥88cm for women and ≥102cm for men |
| **Triglycerides** | ≥150mg/dl or drug treatment for elevated triglycerides (fibrates, nicotinic acid, high dose of omega-3 fatty acids) | ≥150mg/dl or drug treatment for elevated triglycerides (fibrates, nicotinic acid, high dose of omega-3 fatty acids) | ≥150mg/dl or drug treatment for elevated triglycerides (fibrates, nicotinic acid) |
| **HDL cholesterol** | <40mg/dl in males and <50mg/dl in females or drug treatment for reduced HDL levels (fibrates, nicotinic acid) | <40mg/dl in males and <50mg/dl in females or drug treatment for reduced HDL levels (fibrates, nicotinic acid) | <40mg/dl in males and <50mg/dl in females or drug treatment for reduced HDL levels (fibrates, nicotinic acid) |
| **Blood pressure** | SBP ≥130 and/or DBP ≥85 mm Hg or antihypertensive drug treatment | SBP ≥130 and/or DBP ≥85 mm Hg or antihypertensive drug treatment | SBP ≥130 and/or DBP ≥85 mm Hg or antihypertensive drug treatment |
| **Fasting blood glucose** | ≥100mg/dl or drug treatment of elevated glucose | ≥100 mg/dl or previously diagnosed diabetes | ≥100mg/dl or drug treatment of elevated glucose |

JIS: Joint Interim Statement; IDF: International Diabetes Federation; NCEP-ATPIII: National Cholesterol Education Program Adult Treatment Panel III; HDL: High-density lipoprotein; SBP: Systolic blood pressure; DBP: Diastolic blood pressure

**Supplementary Table 2. Univariate determinants for the presence of metabolic syndrome in APS patients based on JIS, IDF and modified NCEP-ATPIII criteria**

| **Parameters** | **JIS criteria** | **IDF criteria** | **Modified NCEP- ATPIII criteria** |
| --- | --- | --- | --- |
| **Age (years)** | OR 1.032 (95% CI 1.000,1.065), p=0.049 | OR 1.029 (95% CI 0.998,1.062), p=0.070 | OR 1.029 (95% CI 0.996,1.063), p=0.087 |
| **Female sex** | OR 1.089 (95% CI 0.470,2.526), p=0.842 | OR 1.249 (95% CI 0.527,2.964), p=0.614 | OR 0.824 (95% CI 0.349,1.947), p=0.659 |
| **SLE-related APS (using primary APS as reference category)** | OR 0.918 (95% CI 0.419,2.016), p=0.832 | OR 0.975 (95% CI 0.442,2.149), p=0.950 | OR 0.890 (95% CI 0.388,2.043), p=0.784 |
| **Disease duration (years)** | OR 1.004 (95% CI 0.957,1.052), p=0.881 | OR 1.002 (95% CI 0.955,1.051), p=0.929 | OR 0.824 (95% CI 0.981 (95% CI 0.931,1.034), p=0.485 |
| **Family history of CAD** | OR 0.591 (95% CI 0.160,2.188), p=0.431 | OR 0.616 (95% CI 0.166,2.284), p=0.469 | OR 0.735 (95% CI 0.197,2.740), p=0.646 |
| **Smoking (pack-years)** | OR 1.023 (95% CI 1.001,1.044), p= 0.037 | OR 1.024 (95% CI 1.003, 1.046), p=0.026 | OR 1.025 (95% CI 1.003,1.047), p=0.025 |
| **Exercise (min/week)** | OR 0.997 (95% CI 0.993,1.000), p=0.060 | OR 0.997 (95% CI 0.993,1.000), p=0.074 | OR 0.997 (95% CI 0.994,1.000), p=0.112 |
| **Cholesterol (mg/dl)** | OR 1.013 (95% CI 1.003,1.024), p=0.014 | OR 1.014 (95% CI 1.004,1.0250, p=0.008 | OR 1.012 (95% CI 1.001,1.023), p=0.029 |
| **LDL (mg/dl)** | OR 1.015 (95% CI 1.003,1.027), p=0.016 | OR 1.016 (95% CI 1.004,1.028), p=0.010 | OR 1.015 (95% CI 1.002,1.027), p=0.021 |
| **Statins, current use** | OR 2.448 (95% CI 1.008,5.940), p=0.048 | OR 2.578 (95% CI 1.059,6.278), p=0.037 | OR 2.091 (95% CI 0.829, 5.276), p=0.118 |
| **High/very-high risk SCORE class (using low/moderate risk SCORE class as reference category)** | OR 3.379 (95% CI 1.095,10.426), p=0.034 | OR 3.536 (95% CI 1.144,10.930), p=0.028 | OR 3.060 (95% CI 0.973,9.619), p=0.056 |
| **CRP (mg/l)** | OR 1.071 (95% CI 1.006,1.141), p=0.033 | OR 1.073 (95% CI 1.007,1.144), p=0.029 | OR 1.061 (95% CI 0.997,1.129), p=0.060 |
| **UA (mg/dl)** | OR 1.560 (95% CI 1.206,2.018), p= 0.001 | OR 1.552 (95% CI 1.200,2.007), p=0.001 | OR 1.654 (95% CI 1.258,2.175), p=0.000 |
| **CKD** | OR 3.226 (95% CI 0.876,11.877), p=0.078 | OR 3.367 (95% CI 0.913,12.414), p=0.068 | OR 2.568 (95% CI 0.676,9.750), p=0.166 |
| **Arterial thrombosis** | OR 2.468 (95% CI 1.118,5.452), p=0.025 | OR 2.739 (95% CI 1.218,6.157), p=0.015 | OR 2.103 (95% CI 0.921,4.803), p=0.078 |
| **Venous thrombosis** | OR 0.745 (95% CI 0.348,1.596), p=0.449 | OR 0.695 (95% CI 0.322,1.499), p=0.353 | OR 0.711 (95% CI 0.319,1.584), p=0.404 |
| **Obstetric APS *** | OR 0.690 (95% CI 0.265,1.792), p=0.446 | OR 0.690 (95% CI 0.265,1.792), p=0.446 | OR 0.619 (95% CI 0.217,1.768), p=0.371 |
| **Hydroxychloroquine, current use** | OR 0.396 (95% CI 0.170,0.923), p=0.032 | OR 0.345 (95% CI 0.143,0.828), p=0.017 | OR 0.433 (95% CI 0.178,1.052), p=0.065 |
| **Hydroxychloroquine use duration (months)** | OR 0.997 (95% CI 0.990,1.004), p=0.395 | OR 0.997 (95% CI 0.990,1.004), p=0.441 | OR 0.996 (95% CI 0.988,1.004), p=0.275 |
| **Cortisone, current use** | OR 1.750 (95% CI 0.788,3.885), p=0.169 | OR 1.857 (95% CI 0.833,4.143), p=0.130 | OR 2.017 (95% CI 0.880,4.626), p=0.097 |
| **Cumulative prednisone dose (mg)** | OR 0.9999961 (95% CI 0.9999695,1.000023), p=0.775 | OR 0.999973 (95% CI 0.9999709,1.000024), p=0.841 | OR 0.9999979 (95% CI 0.9999704,1.000025), p=0.880 |
| **Aspirin, current use** | OR 0.543 (95% CI 0.242,1.217), p=0.138 | OR 0.575 (95% CI 0.256,1.293), p=0.181 | OR 0.509 (95% CI 0.215,1.206), p=0.125 |
| **Anticoagulants, current use** | OR 3.507 (95% CI 1.145,10.742), p=0.028 | OR 3.351 (95% CI 1.093,10.280), p=0.034 | OR 2.769 (95% CI 0.897,8.554), p=0.077 |
| **Immunosuppressive drugs, current use** | OR 0.566 (95% CI 0.179,1.791), p=0.333 | OR 0.591 (95% CI 0.186,1.872), p=0.371 | OR 0.710 (95% CI 0.222,2.265), p=0.562 |
| **Anti-cardiolipin IgG positivity** | OR 1.088 (95% CI 0.489,2.423), p=0.836 | OR 1.028 (95% CI 0.460,2.297), p=0.947 | OR 1.154 (95% CI 0.494,2.694), p=0.741 |
| **High-titre anti-cardiolipin IgG** | OR 1.375 (95% CI 0.607,3.112), p=0.445 | OR 1.217 (95% CI 0.530,2.795), p=0.644 | OR 1.270 (95% CI 0.536,3.007), p=0.587 |
| **Anti-cardiolipin IgM positivity** | OR 0.674 (95% CI 0.315,1.442), p=0.309 | OR 0.724 (95% CI 0.336,1.559), p=0.409 | OR 0.712 (95% CI 0.320,1.585), p=0.405 |
| **High-titre anti-cardiolipin IgM** | OR 1.019 (95% CI 0.409,2.535), p=0.968 | OR 1.069 (95% CI 0.429,2.668), p=0.866 | OR 0.816 (95% CI 0.301,2.210), p=0.689 |
| **Anti-β2-glycoprotein I IgG positivity** | OR 0.914 (95% CI 0.427,1.957), p=0.817 | OR 0.840 (95% CI 0.389,1.815), p=0.657 | OR 0.953 (95% CI 0.428,2.121), p=0.906 |
| **High-titre anti-β2-glycoprotein I IgG** | OR 1.870 (95% CI 0.791,4.421), p=0.154 | OR 1.619 (95% CI 0.675,3.885), p=0.281 | OR 1.636 (95% CI 0.662,4.047), p=0.286 |
| **Anti-β2-glycoprotein I IgM positivity** | OR 1.692 (95% CI 0.788,3.633), p=0.177 | OR 1.819 (95% CI 0.841,3.935), p=0.129 | OR 1.819 (95% CI 0.841,3.935), p=0.129 |
| **High-titre anti-β2-****glycoprotein I IgM** | OR 2.063 (95% CI 0.733,5.807), p=0.170 | OR 2.159 (95% CI 0.765,6.091), p=0.146 | OR 1.435 (95% CI 0.469,4.392), p=0.527 |
| **Lupus anticoagulant positivity** | OR 0.952 (95% CI 0.418,2.170), p=0.907 | OR 1.081 (95% CI 0.466,2.510), p=0.856 | OR 0.862 (95% CI 0.365,2.032), p=0.734 |
| **High-titre antiphospholipid antibody** | OR 1.188 (95% CI 0.557,2.534), p=0.657 | OR 1.099 (95% CI 0.511,2.361), p=0.810 | OR 1.085 (95% CI 0.488,2.409), p=0.842 |
| **Triple antiphospholipid antibody positivity** | OR 1.067 (95% CI 0.498,2.287), p=0.868 | OR 1.142 (95% CI 0.529,2.462), p=0.736 | OR 1.104 (95% CI 0.495,2.461), p=0.809 |
| **Atherosclerotic plaques at any site** | OR 4.337 (95% CI 1.927, 9.763), p=0.000 | OR 3.981 (95% CI 1.766, 8.974), p=0.001 | OR 4.015 (95% CI 1.725,9.344), p=0.001 |
| **Atherosclerotic plaques at the carotid arteries** | OR 4.875 (95% CI 2.091,11.363), p=0.000 | OR 4.333 (95% CI 1.861,10.091), p=0.001 | OR 3.947 (95% CI 1.664,9.365), p=0.002 |
| **Atherosclerotic plaques at the femoral arteries** | OR 1.833 (95% CI 0.763,4.403), p=0.175 | OR 1.935 (95% CI 0.803,4.662), p=0.141 | OR 1.980 (95% CI 0.801,4.891), p=0.139 |
| **Total number of atherosclerotic plaques** | OR 1.588 (95% CI 1.173,2.150), p=0.003 | OR 1.587 (95% CI 1.172,2.150), p=0.003 | OR 1.551 (95% CI 1.141,2.107), p=0.005 |

* Univariable analysis performed only in female patients

# The following parameters: body-mass index, waist circumference, abdominal obesity, obesity, blood pressure (systolic and diastolic), hypertension and current use of antihypertensives, high-density lipoprotein and triglycerides levels, dyslipidaemia and atherogenic dyslipidaemia and glucose levels are not included in the table, as they are part of metabolic syndrome diagnostic criteria

CI: Confidence intervals; JIS: Joint Interim Statement; IDF: International Diabetes Federation; NCEP-ATPIII: National Cholesterol Education Program Adult Treatment Panel III; APS: Antiphospholipid syndrome; SLE: Systemic lupus erythematosus; CAD: Coronary artery disease; LDL: Low-density lipoprotein; SCORE: Systemic Coronary Risk Evaluation; CRP: C-reactive protein; UA: Uric acid; CKD: Chronic kidney disease
